# A case–control comparison of acute-phase peripheral blood gene expression in participants diagnosed with minor ischaemic stroke or stroke mimics

**DOI:** 10.1186/s40246-023-00551-y

**Published:** 2023-11-25

**Authors:** Joseph V. Moxon, Andrew Calcino, Ann-Katrin Kraeuter, James Phie, Georgina Anderson, Glenys Standley, Cindy Sealey, Rhondda E. Jones, Matt A. Field, Jonathan Golledge

**Affiliations:** 1 Queensland Research Centre for Peripheral Vascular Disease, College of Medicine and Dentistry, James Cook University, Townsville, QLD 4811 Australia; 2 Australian Institute of Tropical Health and Medicine, James Cook University, Townsville, QLD 4811 Australia; 3 Centre for Tropical Bioinformatics and Molecular Biology, James Cook University, Townsville, QLD 4811 Australia; 4 Faculty of Health and Life Sciences, Psychology, Northumbria University, Newcastle Upon Tyne, UK; 5 Research Division, James Cook University, Townsville, QLD 4811 Australia; 6 Tropical Australian Academic Health Centre, Townsville, QLD 4811 Australia; 7 Immunogenomics Laboratory, Garvan Institute of Medical Research, Darlinghurst, NSW 2010 Australia; 8 Menzies School of Health Research, Darwin, NT 0811 Australia; 9 Department of Vascular and Endovascular Surgery, Townsville University Hospital, Townsville, QLD 4811 Australia

**Keywords:** Ischaemic stroke, Stroke mimics, RNA-seq, miRNA, Peripheral blood gene expression

## Abstract

**Background:**

Past studies suggest that there are changes in peripheral blood cell gene expression in response to ischaemic stroke; however, the specific changes which occur during the acute phase are poorly characterised. The current study aimed to identify peripheral blood cell genes specifically associated with the early response to ischaemic stroke using whole blood samples collected from participants diagnosed with ischaemic stroke (*n* = 29) or stroke mimics (*n* = 27) following emergency presentation to hospital. Long non-coding RNA (lncRNA), mRNA and micro-RNA (miRNA) abundance was measured by RNA-seq, and the consensusDE package was used to identify genes which were differentially expressed between groups. A sensitivity analysis excluding two participants with metastatic disease was also conducted.

**Results:**

The mean time from symptom onset to blood collection was 2.6 h. Most strokes were mild (median NIH stroke scale score 2.0). Ten mRNAs (all down-regulated in samples provided by patients experiencing ischaemic stroke) and 30 miRNAs (14 over-expressed and 16 under-expressed in participants with ischaemic stroke) were significantly different between groups in the whole cohort and sensitivity analyses. No significant over-representation of gene ontology categories by the differentially expressed genes was observed. Random forest analysis suggested a panel of differentially expressed genes (ADGRG7 and miRNAs 96, 532, 6766, 6798 and 6804) as potential ischaemic stroke biomarkers, although modelling analyses demonstrated that these genes had poor diagnostic performance.

**Conclusions:**

This study provides evidence suggesting that the early response to minor ischaemic stroke is predominantly reflected by changes in the expression of miRNAs in peripheral blood cells. Further work in independent cohorts particularly in patients with more severe stroke is needed to validate these findings and investigate their clinical relevance.

**Supplementary Information:**

The online version contains supplementary material available at 10.1186/s40246-023-00551-y.

## Introduction

Each year an estimated 12 million strokes occur across the world, approximately 70% of which are caused by cerebral ischaemia (acute ischaemic stroke) [1]. Prior studies have demonstrated the feasibility of using RNA-sequencing (RNA-seq) to identify differences in the genes expressed by peripheral blood cells collected from individuals with or without ischaemic stroke, and have suggested a range of RNAs as potential diagnostic or prognostic markers. Translating these findings to clinical practice is, however, complicated due to marked differences in the design of independent studies (reviewed in [2–4]). For example, most previous RNA-seq-based investigations have compared peripheral blood gene expression between groups of patients and healthy volunteers which does not reflect the clinical need to discriminate patients experiencing ischaemic stroke from those with unrelated neurological symptoms [5–9]. Secondly, many prior studies have analysed blood samples collected from patients many hours after ischaemic stroke onset, by which time brain infarction is usually established and currently indicated deadlines for administering front-line stroke therapies have elapsed [5–13]. Thus, there is currently limited understanding of the acute-phase changes in peripheral blood cell gene expression which are specifically associated with ischaemic stroke onset. The current study aimed to address this gap in knowledge by identifying differences in the expression of mRNAs, long non-coding RNAs (lncRNA) and micro-RNAs (miRNA) in peripheral blood samples collected from patients diagnosed with ischaemic stroke, or stroke mimics following emergency presentation to hospital.

## Results

### Participant characteristics

**Fig. 1:**
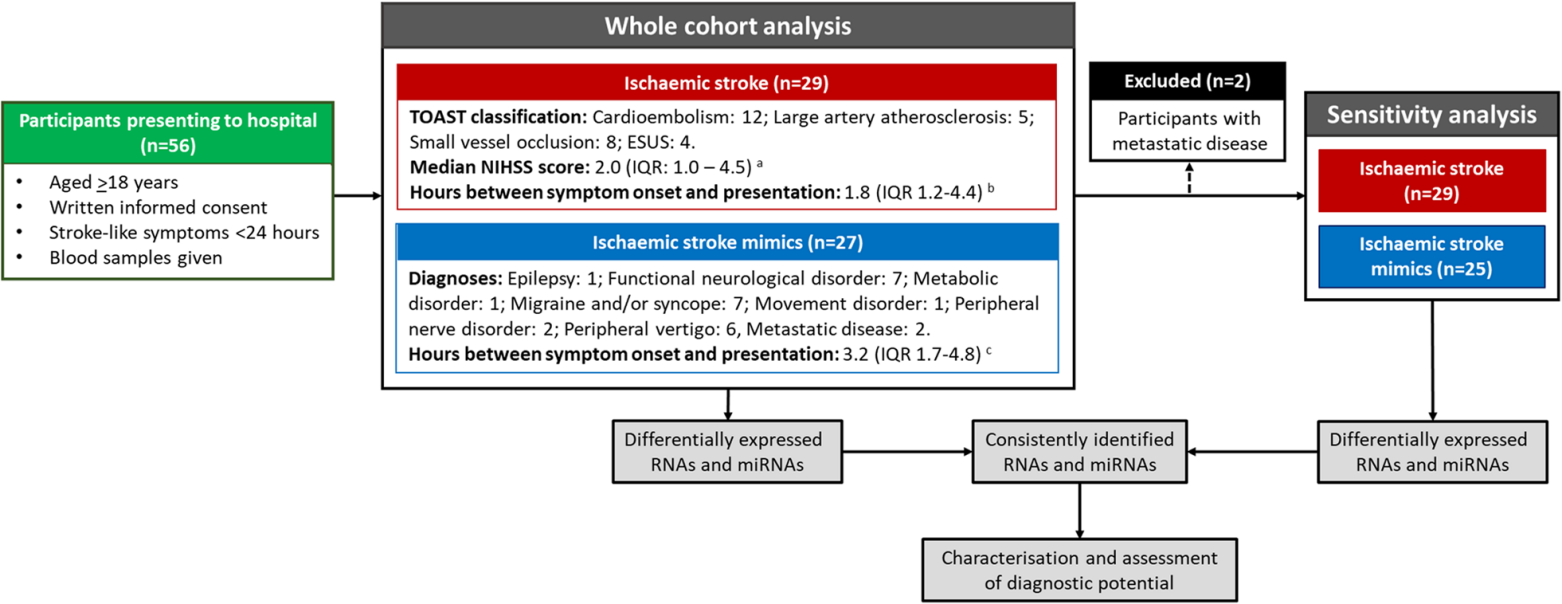
Participant flow for the current study. ESUS: Embolic stroke of Unknown Source. **A** Based on 22 observations (75.9% of the group). **B** Based on 26 observations (89.7% of the group). **C** Based on 22 observations (84.6% of the group)

Fifty-six participants were initially recruited to this study (Table 1, Fig. 1). Twenty-nine participants received a confirmed ischaemic stroke diagnosis, the majority of which arose from cardioembolism or small vessel occlusion (collectively accounting for ~ 70% of all presentations). Twenty-seven participants diagnosed with stroke mimics were recruited. Stroke-like symptoms in this groups arose from a heterogeneous mix of conditions (Fig. 1). Median time from symptom onset to hospital presentation for the cohort was ~ 2.5 h (range 0–23 h based on 49 observations) and did not differ significantly between groups. Participants who received a stroke diagnosis were significantly more likely to have had a previous stroke than those with stroke mimics. The groups were otherwise well matched for cardiovascular risk factors and prescribed medications (Table 1).

**Table 1:** Characteristics of participants included in the analyses

Characteristic	Cohort (*n* = 56)	Ischaemic stroke (*n* = 29)	Stroke mimic (*n* = 27)	*p* value
*Part 1: analysis including whole cohort*				
Age	66.8 (58.8–74.7)	66.8 (58.8–74.7)	67.0 (59.7–74.9)	0.768
BMI	28.2 (25.3–31.2) [3]	29.4 (25.7–33.2)	27.7 (24.1–30.0) [3]	0.110
Symptom duration*	2.6 (1.2–4.7) [7]	1.8 (1.2–4.4) [3]	3.2 (1.7–4.8) [4]	0.336
Male sex	28 (50.0%)	15 (51.7%)	13 (48.1%)	1.000
NIHSS score at presentation	NA	2.0 (1.0–4.5) [7]	NA	NA
*Smoking history*				
Never smoked	23 (41.1%)	12 (41.4%)	11 (40.7%)	0.832
Ex-smoker	23 (41.1%)	11 (37.9%)	12 (44.4%)	
Current smoker	10 (17.9%)	6 (20.7%)	4 (14.8%)	
*History of*				
Hypertension	38 (67.9%)	20 (69.0%)	18 (66.7%)	1.000
CHD	20 (35.7%)	12 (41.4%)	8 (29.6%)	0.412
TIA	14 (25.0%)	8 (27.6%)	6 (22.2%)	0.761
Stroke	25 (44.6%)	18 (62.1%)	7 (25.9%)	0.008
Diabetes	10 (17.9%)	6 (20.7%)	4 (14.8%)	0.731
*Prescription for*				
Aspirin	14 (25.0%)	7 (24.1%)	7 (25.9%)	1.000
Other antiplatelet agents	6 (10.7%)	3 (10.3%)	3 (11.1%)	1.000
Calcium channel blockers	9 (16.1%)	4 (13.8%)	5 (18.5%)	0.725
Beta blockers	13 (23.2%)	6 (20.7%)	7 (25.9%)	0.756
ACE inhibitors	9 (16.1%)	6 (20.7%)	3 (11.1%)	0.472
Statins	21 (37.5%)	12 (41.4%)	9 (33.3%)	0.589

Characteristic	Cohort (*n* = 54)	Ischaemic stroke (*n* = 29)	Stroke mimic (*n* = 25)	*p* value
*Part 2: analysis excluding participants with metastatic disease*				
Age	66.8 (58.5–74.8)	66.8 (58.5–74.8)	67.0 (59.6–75.2)	0.835
BMI	28.0 (25.2–31.3) [2]	29.4 (25.7–33.2)	27.7 (24.0–30.1) [2]	0.098
Symptom duration (hours)*	2.6 (1.3–4.7) [7]	1.8 (1.2–4.4) [3]	3.2 (1.8–4.7) [4]	0.314
Male sex	26 (48.1%)	15 (51.7%)	11 (44.0%)	0.769
NIHSS score at presentation	NA	2.0 (1.0–4.5) [7]	NA	NA
*Smoking history*				
Never smoked	23 (42.6%)	12 (41.4%)	11 (44.0%)	0.718
Ex-smoker	22 (40.7%)	11 (37.9%)	11 (44.0%)	
Current smoker	9 (16.7%)	6 (20.7%)	3 (12.0%)	
*History of*				
Hypertension	36 (66.7%)	20 (69.0%)	16 (64.0%)	0.777
CHD	20 (37.0%)	12 (41.4%)	8 (32.0%)	0.576
TIA	14 (25.9%)	8 (27.6%)	6 (24.0%)	1.000
Stroke	23 (42.6%)	18 (62.1%)	5 (20.0%)	0.002
Diabetes	10 (18.5%)	`6 (20.7%)	4 (16.0%)	0.736
*Prescription for*				
Aspirin	13 (24.1%)	7 (24.1%)	6 (24.0%)	1.000
Other antiplatelet agents	6 (11.1%)	3 (10.3%)	3 (12.0%)	1.000
Calcium channel blockers	9 (16.7%)	4 (13.8%)	5 (20.0%)	0.718
Beta blockers	13 (24.1%)	6 (20.7%)	7 (28.0%)	0.751
ACE inhibitors	9 (16.7%)	6 (20.7%)	3 (12.0%)	0.480
Statins	20 (37.0%)	12 (41.4%)	8 (32.0%)	0.576

*NIHSS* National Institutes of Health Stroke Severity, *CHD* Coronary heart disease, *TIA* Transient Ischaemic Attack, *NA* Not applicable. Numbers in square brackets refer to the number of missing datapoints for that variable

*Refers to hours between symptom onset and hospital presentation

### Identifying inter-group differences in the expression of peripheral blood cell lncRNAs, mRNAs and miRNAs

**Fig. 2:**
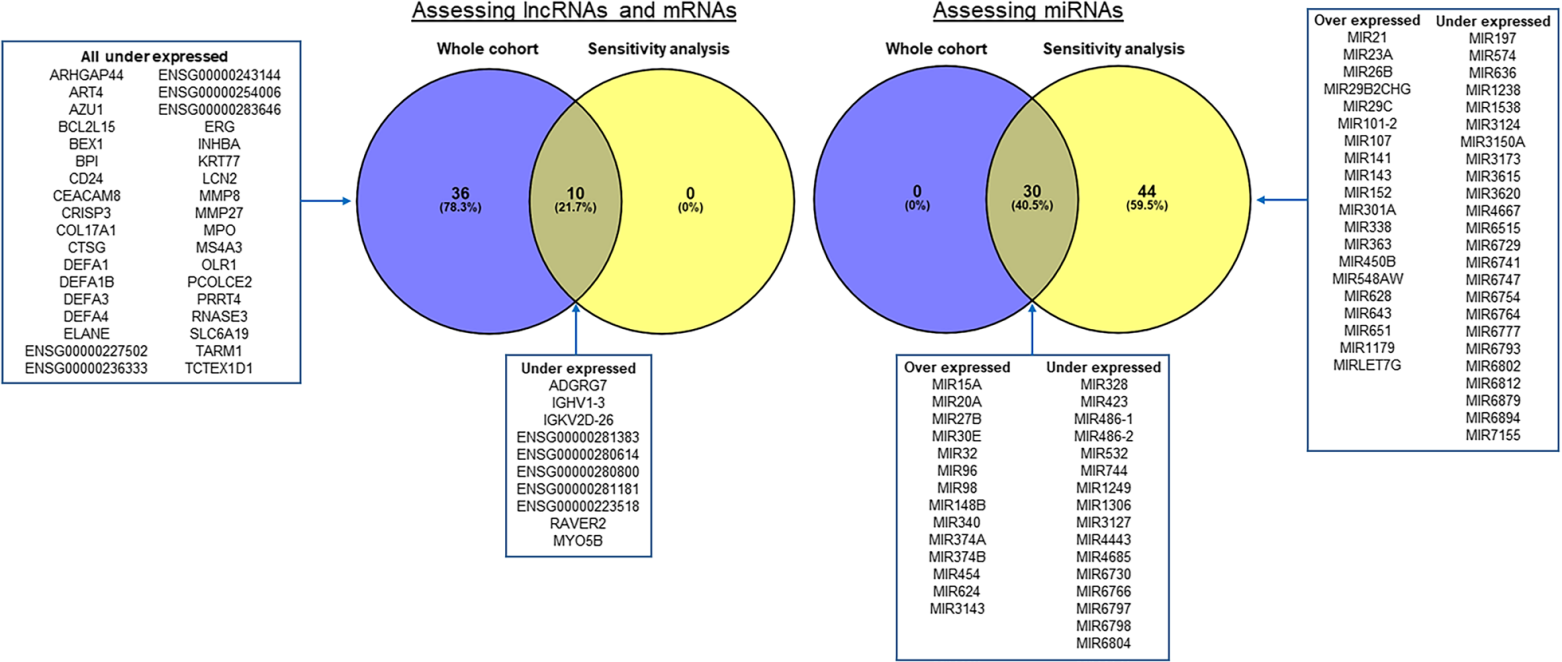
Differentially expressed lncRNAs, mRNAs and micro-RNAs identified in whole cohort and sensitivity analyses

A total of 48,432 unique RNA transcripts were detected in all patients during the RNA-seq experiments. Forty-six mRNA transcripts (including ten unannotated genes) and 30 miRNAs showed significant differences in expression between groups (Additional file 1: Supplements 1 and 2). Examination of the differentially expressed protein-coding RNAs revealed marked over-expression of genes associated with neutrophil degranulation by a single participant in the stroke mimic group, for whom neurological symptoms were attributed to complications of metastatic disease (Additional file 1: Supplement 3). Given high potential for these outliers to bias results, a sensitivity analysis excluding two participants with known metastatic disease was conducted (Table 1). The sensitivity analysis identified significant inter-group differences in the expression of 10 mRNAs RNAs and 74 miRNAs (Additional file 1: Supplements 4 and 5). Forty genes (10 mRNAs and 30 miRNAs) were identified as differentially expressed in both the whole cohort and sensitivity analyses with similar magnitudes of inter-group differences and were shortlisted for further characterisation (Figs. 1, 2 and Table 2). No associations between expression of the shortlisted mRNAs or miRNAs with stroke severity (NIHSS score), symptom duration or suspected stroke aetiology (TOAST classification) were observed (Additional file 1: Supplement 6).

**Table 2:** Differentially expressed mRNAs and miRNAs identified in whole cohort and sensitivity analyses

Gene ID	Gene symbol [family]	Difference between groups (whole cohort analysis)			Difference between groups (sensitivity analysis)		
		LogFC	LogFC sd	*p* value	LogFC	LogFC sd	*p* value
*Long RNAs*							
ENSG00000144820	ADGRG7	− 3.08	1.97	3.02E−08	− 3.22	1.96	6.90E−09
ENSG00000211935	IGHV1-3	− 2.26	1.45	5.23E−04	− 2.35	1.42	6.14E−04
ENSG00000211623	IGKV2D-26	− 3.18	2.27	1.76E−03	− 3.23	2.32	6.75E−03
ENSG00000281383	ENSG00000281383	− 1.86	1.46	3.67E−03	− 1.96	1.49	8.02E−03
ENSG00000167306	MYO5B	− 1.09	0.29	1.04E−02	− 1.21	0.27	8.24E−03
ENSG00000280800	ENSG00000280800	− 1.73	1.35	6.73E−03	− 1.82	1.38	9.80E−03
ENSG00000280614	ENSG00000280614	− 1.73	1.35	6.73E−03	− 1.82	1.38	9.80E−03
ENSG00000281181	ENSG00000281181	− 1.73	1.35	6.73E−03	− 1.82	1.38	9.80E−03
ENSG00000223518	ENSG00000223518	− 2.97	2.19	8.26E−03	− 3.06	2.24	1.85E−02
ENSG00000162437	RAVER2	− 0.99	0.34	8.26E−03	− 1.01	0.35	2.59E−02
*Micro-RNAs*							
ENSG00000198974	MIR30E [mir-30]	0.32	0.07	9.00E−04	0.35	0.09	4.36E−05
ENSG00000276830	MIR6730	− 0.52	0.06	1.41E−02	− 0.6	0.08	8.45E−04
ENSG00000284464	MIR1306 [mir-1306]	− 0.38	0.07	1.43E−02	− 0.44	0.09	1.11E−03
ENSG00000265565	MIR3143	0.41	0.08	1.06E−02	0.44	0.09	2.34E−03
ENSG00000283450	MIR486-2 [mir-486]	− 0.43	0.08	1.43E−02	− 0.47	0.09	3.47E−03
ENSG00000276926	MIR6797	− 0.55	0.06	1.43E−02	− 0.6	0.07	3.47E−03
ENSG00000221598	MIR1249 [mir-1249]	− 0.52	0.08	1.68E−02	− 0.56	0.09	5.38E−03
ENSG00000207948	MIR328 [mir-328]	− 0.33	0.07	2.95E−02	− 0.37	0.08	7.56E−03
ENSG00000274705	MIR486-1 [mir-486]	− 0.35	0.08	3.10E−02	− 0.39	0.09	7.62E−03
ENSG00000264610	MIR4685	− 0.39	0.07	1.99E−02	− 0.41	0.08	7.62E−03
ENSG00000275101	MIR6766	− 0.42	0.08	3.59E−02	− 0.48	0.09	7.62E−03
ENSG00000273898	MIR6798	− 0.39	0.09	4.95E−02	− 0.47	0.1	7.62E−03
ENSG00000283935	MIR423 [mir-423]	− 0.33	0.07	1.99E−02	− 0.34	0.08	7.81E−03
ENSG00000207758	MIR532 [mir-188]	− 0.25	0.07	3.61E−02	− 0.28	0.08	7.81E−03
ENSG00000199168	MIR374A [mir-374]	0.6	0.07	2.02E−02	0.66	0.08	8.20E−03
ENSG00000207952	MIR624 [mir-624]	0.55	0.07	1.93E−02	0.57	0.08	1.03E−02
ENSG00000283785	MIR15A [mir-15]	0.53	0.09	4.09E−02	0.58	0.1	1.04E−02
ENSG00000199122	MIR148B [mir-148]	0.21	0.07	4.02E−02	0.23	0.08	1.04E−02
ENSG00000212027	MIR374B [mir-374]	0.49	0.07	4.09E−02	0.54	0.08	1.04E−02
ENSG00000275519	MIR6804	− 0.43	0.07	4.45E−02	− 0.5	0.08	1.05E−02
ENSG00000207698	MIR32 [mir-32]	0.92	0.07	4.42E−02	1.01	0.08	1.34E−02
ENSG00000271886	MIR98 [let-7]	0.5	0.08	2.75E−02	0.49	0.09	1.34E−02
ENSG00000283762	MIR20A [mir-17]	0.55	0.12	4.76E−02	0.59	0.13	1.37E−02
ENSG00000198995	MIR340 [mir-340]	0.27	0.07	4.76E−02	0.29	0.08	1.37E−02
ENSG00000211514	MIR454 [mir-454]	0.32	0.08	4.76E−02	0.33	0.09	1.37E−02
ENSG00000199158	MIR96 [mir-96]	0.56	0.09	4.09E−02	0.59	0.1	1.51E−02
ENSG00000265483	MIR4443	− 0.8	0.19	3.32E−02	− 0.73	0.22	1.87E−02
ENSG00000207864	MIR27B [mir-27]	0.38	0.07	4.76E−02	0.39	0.08	2.22E−02
ENSG00000266297	MIR744 [mir-744]	− 0.26	0.07	4.14E−02	− 0.26	0.08	2.42E−02
ENSG00000264157	MIR3127 [mir-3127]	− 0.3	0.08	4.81E−02	− 0.3	0.09	2.57E−02

miRNA gene family assignment based on miRbase annotation. Log FC: Fold change in transcript expression in participants with strokes, compared to stroke mimics. Std Dev: Standard deviation of fold change. *p* value refers to false discovery rate corrected ‘P-union’ *p* value reported by consensusDE. *NA* not annotated. Annotations in square brackets refer to the miRNA family to which the identified miRNAs belong

### Characterising differentially expressed mRNAs and miRNAs

Fourteen miRNAs, including two MIR374 isoforms (MIR374a and b), were significantly over-expressed by participants with ischaemic stroke compared to stroke mimics. The remaining 16 differentially expressed miRNAs including two members of the MIR486 family (MIR486-1 and MIR486-2) were less abundant in the participants with ischaemic stroke than those with stroke mimics. All 10 differentially expressed long RNAs were at lower abundance in the participants with ischaemic stroke than those with stroke mimics. Five of the differentially expressed mRNAs were unannotated. Of these four (ENSG00000281383, ENSG00000280614, ENSG00000280800 and ENSG00000281181) appeared to constitute a cluster of single exon orthologues of the YAM1 long non-coding RNA (GeneCards.org) and showed near-identical expression (correlation coefficients > 0.99). The remaining unannotated RNA was suggested to be a pseudogene (ENSG00000223518, Casein Kinase 1 Alpha 1 Pseudogene 1, GeneCards.org). Collectively these unannotated genes were considered artefacts. Analysis of the five annotated mRNAs revealed no significant enrichment for any gene ontology category (Webgestalt), and no predicted interactions between gene products (Cytoscape). Database searches highlighted MYO5B and RAVER2 as suggested targets of several of the differentially expressed miRNAs; however, no correlation in the expression of these mRNAs and the targeting miRNAs was observed (Additional file 1: Supplement 7).

### Using RNA data to predict ischaemic stroke diagnosis

**Fig. 3:**
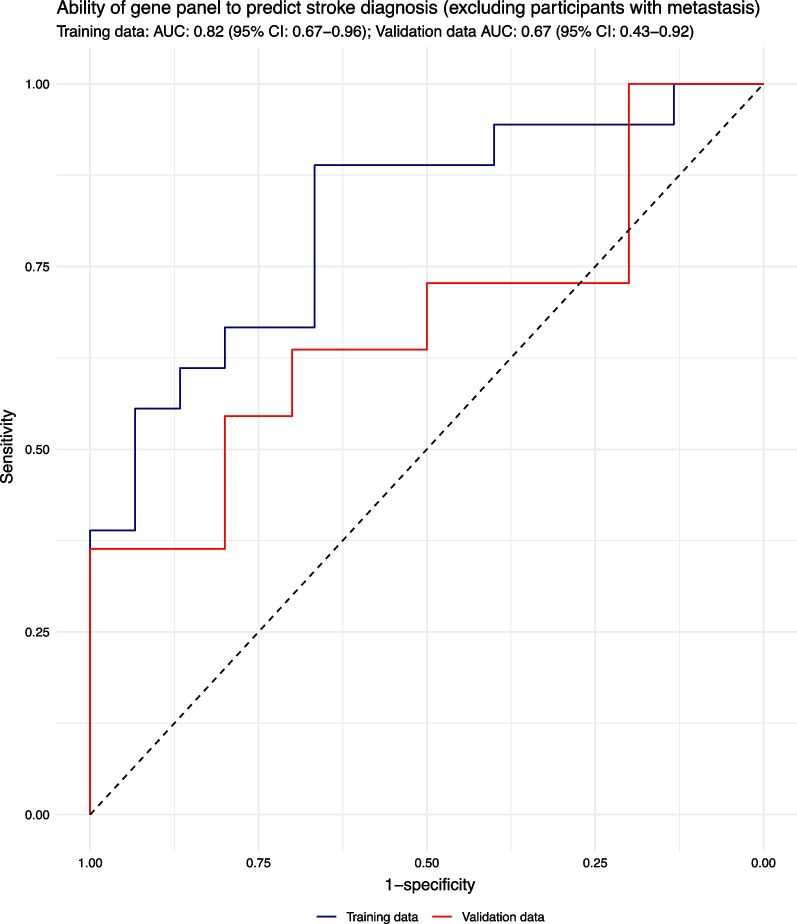
ROC curves showing the diagnostic performance of a panel comprising ADGRG7 and miRs 96, 532, 6766, 6798 and 6804 to predict ischaemic stroke diagnosis in training and validation sets. Note participants with metastatic disease are excluded from this analysis

The 40 differentially expressed mRNAs and miRNAs were subjected to 3 machine learning approaches (partial least squares regression, root mean standard error regression and random forest) to develop classifier models to distinguish patients experiencing ischaemic stroke from those with stroke mimics. All models performed well on the training data evidenced by a classification accuracy ≥ 85%, with random forest-based models showing the best stratification performance (Additional file 1: Supplement 8). Examining variable importance plots generated by the random forest models highlighted six RNAs (ADGRG7 and miRs 96, 532, 6766, 6798 and 6804) with the greatest discriminant power evidenced by high mean decrease in accuracy scores across all 5 random forest models (Additional file 1: Supplement 9). A logistic regression model incorporating these six RNAs showed moderate potential to distinguish participants with ischaemic stroke from those with stroke mimics in the training dataset (classification accuracy 75.8% (95%CI: 57.7–88.9%, area under the ROC curve 0.88). When applied to the validation dataset, classification performance dropped to 47.6% (25.7–70.2%), with a corresponding area under the ROC curve of 0.67 (Fig. 3).

## Discussion

The current study represents the first application of RNA-seq to characterise acute-phase differences in peripheral blood cell gene expression between groups of participants experiencing ischaemic stroke or stroke mimics. Forty genes were observed to be consistently differentially expressed following whole cohort and sensitivity analyses suggesting that these genes may represent the early response to cerebral ischaemia, as opposed to a generic reaction to unrelated neurological disturbances. Findings suggest that this response is most pronounced at the miRNA level; however, interpreting the mechanistic impact of this is complicated as relatively few mRNAs or lncRNAs showed significant inter-group differences in abundance thereby prohibiting detailed pathway analyses. Machine learning approaches identified a panel of six RNAs (one mRNA and five miRNAs) with potential to predict ischaemic stroke presence, although poor performance in validation datasets suggests that these are unlikely to be useful clinical diagnostics.

Prior RNA-seq-based investigations have reported marked differences in miRNA, lncRNA and mRNA abundance in whole blood, serum or plasma samples provided by patients experiencing ischaemic stroke, compared to control groups [5–13]. Inter-group differences in gene expression in the current study were comparatively modest; however, most previous studies have utilised healthy volunteers as a control group which does not reflect clinical need. Relatively few investigations have compared peripheral blood gene expression in patients diagnosed with ischaemic stroke to those experiencing other neurological symptoms [10, 12, 14]. Whilst more closely aligned with the design of the current investigation, these prior studies included patients with more severe stroke and had longer intervals between symptom onset and blood collection which complicates direct comparison. In line with findings from the current cohort, Toor et al. [12] recently reported significant up-regulation of miR-20a in serum samples from patients experiencing ischaemic stroke (*n* = 191) compared to those with transient ischaemic attack (TIA, *n* = 61). Whole blood miR-20a concentration has independently been higher in patients experiencing cardio-embolic stroke compared to ischaemic strokes arising from other sources [15]; however, no difference in miR-20a expression between TOAST categories was observed in the present investigation.

Toor et al. [12] also reported a significantly higher abundance of serum miR-486-3 in ischaemic stroke patients compared to TIA controls. Whilst this was not observed in the current cohort, the expression of two different miR-486 isoforms (miR-486-1 and miR-486-2) was significantly lower in patients with ischaemic stroke than those with stroke mimics. Support for an inverse association of the miR-486 family with ischaemic stroke is provided by prior studies reporting that miR-486-5 abundance is lower in blood samples collected from patients with carotid artery disease compared to controls [16] and is inversely correlated with the severity of carotid artery stenosis [17]. Available evidence suggests a role for the mirR-486 family in regulating wound healing, apoptosis and angiogenesis [18–20], although it remains unclear whether this is beneficial or harmful within the cardiovascular system. Several studies have suggested a protective role for miR-486 in repairing cardiac damage following myocardial ischaemia or infarction [21–24]; however, circulating miR-486 has been conversely suggested to be increased, or decreased in the presence of vascular pathology or following major cardiovascular events [17, 25–27]. No papers specifically detailing the role of miR-486-1 or miR-486-2 could be found, and further investigation into the relevance of reduced expression of these miR-486 family members to ischaemic stroke pathology is needed.

Patients in the ischaemic stroke group significantly over-expressed two miR-374 isoforms (miR-374a and b) when compared to those with stroke mimics, suggesting that this miRNA family may also be important in the response to cerebral ischaemia. No prior reports of an association of the expression of the miR-374 family by peripheral blood cells and clinical ischaemic stroke diagnosis were found; however, miR-374 expression has been conversely reported to be increased and decreased in brains recovered from rodent ischaemic stroke models [28, 29]. A single study has reported that plasma miR-374 expression is predictive of the severity of neonatal hypoxic-ischaemic encephalopathy [30], tentatively supporting a role for this miRNA as a marker for neurological injury, although the relevance of this is unclear.

Few lncRNAs or mRNAs were associated with ischaemic stroke in the current cohort, and 50% of those identified were suggested to be non-coding genes. All differentially expressed mRNAs were significantly lower in abundance in the participants with ischaemic stroke compared to stroke mimics; however, this did not appear to be influenced by the expression of targeting miRNAs. ADGRG7, a membrane-bound G protein-coupled receptor, showed the greatest expression difference between groups, although the physiological significance of this remains unclear. ADGRG7 is an oestrogen-responsive gene implicated in the development and progression of multiple cancer types [31–33], but has not been specifically associated with ischaemic stroke. Similarly, other annotated mRNAs identified in the current study have been previously associated with a range of indications including liver injury (RAVER2, [34, 35]) and intestinal disease (MYO5B, [36, 37]), but have not been directly associated with ischaemic stroke diagnosis.

The findings of the current study must be considered in light of inherent strengths and limitations. The study design was representative of the clinical scenario whereby samples from groups of participants experiencing ischaemic stroke or stroke mimics were compared, although stroke aetiology in the cohort was heterogeneous, likely increasing data complexity. Previous investigators have reported that peripheral blood gene expression varies between TOAST classifications [6, 9–11, 38]. It is possible that pooling participants with ischaemic stroke may have masked molecular differences associated with specific stroke sub-types. Moreover, the narrow range of NIHSS scores within the current cohort may have limited the ability to detect associations between stroke severity and lncRNA, mRNA or miRNA expression. Neurological symptoms in the stroke mimic group were attributed to a range of causes with potential to influence basal gene expression [11, 39–42]. This was particularly true for individuals with metastatic disease in whom aberrant gene expression overtly influenced outcomes of the whole cohort analysis. This was mitigated by conducting a sensitivity analysis excluding these participants and focusing on consistently identified mRNAs and miRNAs. Whilst this strengthened confidence in the association of the shortlisted genes with ischaemic stroke diagnosis, there is potential that pertinent markers may have been excluded. Moreover, this approach also reduced the overall group size thereby limiting the power to identify and test novel genes as potential diagnostics. The decision to analyse whole blood samples may also have influenced the genes identified in the current study as recent reports highlight ischaemic stroke associated changes in gene expression differ between white blood cell types [6, 14]. It is not possible to determine the origin of the differentially expressed genes from the whole blood sample analyses presented here, and the possibility that pertinent markers arising from a specific cell type or blood fraction may have been masked must be considered. Finally, the current study only investigated differences in lncRNA, mRNA and miRNA expression and the relevance of circular RNAs suggested by other researchers to this cohort remains unclear [12, 43–49].

## Conclusions

The current hospital-based study demonstrated that peripheral blood cell gene expression differs significantly between groups of patients experiencing minor ischaemic stroke, or stroke mimics, within hours of symptom onset. Inter-group differences in miRNA expression were more pronounced than for lncRNAs and mRNAs, and data suggest novel associations of the miR-374 and miR-486 family with ischaemic stroke diagnosis. Findings from the current cohort suggest low potential for identified mRNAs and miRNAs to act as clinical diagnostics. Further work in larger independent hospital-based cohorts including participants with more severe ischaemic stroke is needed to validate the association of the observed changes in RNA expression with ischaemic stroke presence, determine their role and relevance to ischaemic stroke pathology and identify markers with stronger diagnostic potential.

## Methods

The current study is reported according to the STROBE guidelines [50].

### Workflow

Figure 1 details the design of this study. Initial analyses compared peripheral blood cell miRNA and RNA profiles of participants with confirmed ischaemic stroke (cases) to those with stroke mimics (controls) including samples collected from all recruited individuals. Observations of marked differences in gene expression by one individual with metastatic disease compared to others in the stroke mimic group led to sensitivity analyses excluding two individuals with this comorbidity. Differentially expressed genes consistently identified in both the whole cohort and sensitivity analyses were selected for further assessment.

### Participants

The current investigation was conducted as part of an ongoing prospective cohort study consecutively recruiting participants aged > 18 years who presented to the Townsville University Hospital, Queensland, Australia, for investigation of stroke-like symptoms of < 24 h duration (recruitment for current study occurred between 2017 and 2020) [51]. To be eligible for inclusion for this investigation, patients had to receive a diagnosis of ischaemic stroke, or a stroke mimic, and provided a high-quality blood sample for RNA analysis. Patients who had received thrombolysis or endovascular clot retrieval prior to sample collection, or who were diagnosed with a transient ischaemic attack, or primary haemorrhagic stroke were not included in this study. Characteristics collected for each participant included sex, age, history of hypertension, diabetes mellitus coronary heart disease (CHD) and prescribed medications as previously described [52, 53]. Details of medications prescribed at the time of presentation were recorded.

### Outcome assessment

Participants were grouped into those diagnosed with ischaemic stroke, or an ischaemic stroke mimic by a consultant neurologist blinded to the results of the RNA analysis. Ischaemic stroke was defined as an acute neurological deficit with evidence of cerebral infarction on cerebral imaging (either computed tomography (CT) and/or magnetic resonance imaging conducted as part of standard care), in line with current guidelines [54]. Stroke mimics were defined as a non-vascular condition presenting with neurological deficits without evidence of brain infarction following assessment of cerebral imaging and clinical history. Ischaemic stroke severity at presentation was estimated by the National Institutes of Health Stroke Severity (NIHSS) score. Ischaemic stroke aetiology was categorised according to the Trial of ORG 10172 in Acute Stroke Treatment (TOAST) criteria [55].

### Blood samples and analysis

Peripheral blood samples were collected into PaxGene tubes (QIAGEN) from all participants at recruitment by the Townsville University Hospital Pathology Department (Pathology Queensland) prior to storing at − 80 °C for later analysis. RNA > 17 bp in length was extracted (PAXgene blood miRNA kit, QIAGEN). Extracted RNA samples were sequenced at the Ramaciotti Centre for Genomics (University of New South Wales, Sydney, Australia). All RNA samples were quality checked for concentration and purity, and integrity (microplate spectrophotometer (Epoch) and TapeStation 4200 (Agilent), respectively). RNA libraries were constructed (miRNA: QIAseq miRNA Library kit with 300 ng input and 16 PCR cycles; total RNA: Truseq stranded total RNA with Ribo-Zero Globin kit using 1000 ng input and 11 PCR cycles) and were quality checked using the ThermoFisher Qubit 4.0 fluorometer (dsDNA HS assay) and the PerkinElmer GX Touch HT (High Sensitivity DNA assay). No samples failed quality control. Libraries were equimolar pooled and sequenced on the NovaSeq 6000 platform.

### Comparing participant characteristics

Normality tests (Shapiro–Wilk test) demonstrated that most continuous variables were not normally distributed. Data are therefore presented as median and inter-quartile range. Inter-group comparisons were performed using the Mann–Whitney U test or Kruskal–Wallis test. Nominal data are presented as count and per cent and were compared between groups using the chi-squared test. Missing data were not imputed.

### Analysis of gene data

Raw read fastq files were run through Trimmomatic [56] to perform QC and remove adapter sequence for the long RNAs. The miRNA library was processed using cutadapt [57] and reads that were ≥ 15 base pairs long and included at least 8 base pairs of adapter were retained. The cleaned lncRNA and mRNA and miRNA reads were aligned to reference genome GRCh38 using STAR aligner software [58] generating gene count and BAM alignment files. Differential gene expression analysis was performed on the lncRNA/mRNA and miRNA data in parallel. Samples were grouped into ‘stroke’ or ‘control’ (stroke mimics), and group differences were identified using the RNA-seq differential expression consensus R-package, consensusDE [59]. LncRNAs, mRNAs and miRNAs showing a *p*-union < 0.05 (corrected for multiple testing using the Benjamini–Hochberg method) after analysis with ConsensusDE were considered to be differentially expressed between the groups. Differentially expressed protein-coding RNAs were searched against the miRDB database to identify potentially interacting miRNAs [60]. The relative relationship in transcript expression was investigated using Spearman’s correlation analysis of normalised fragment counts per million.

Data detailing the abundance of lncRNAs, mRNAs and miRNAs showing significant differences in expression between the groups commonly identified in whole-cohort and sensitivity analyses were partitioned into non-overlapping training and validation sets (60:40 splits respectively) ensuring an equal proportion of participants experiencing ischaemic stroke in each dataset (ischaemic stroke prevalence 54.5% and 52.4% in the training and validation datasets, respectively). Participants with metastatic disease were excluded due to a high potential to bias analysis through atypical gene expression. Training data were used to generate models to predict ischaemic stroke presence using partial least squares and root mean-square error regression (both employing fivefold cross-validation) using the caret and glmnet R packages [61, 62] and random forest (five separate analyses including 1000–3000 trees increasing in 500 tree increments) employing the R randomForest package [63]. The ability for the machine learning approaches to classify patients according to the presence or absence of ischaemic stroke was assessed using confusion matrices; the machine learning approach showing the highest classification performance on the training data was selected to identify RNA markers with the greatest discriminant ability. A binary logistic regression model incorporating these RNA markers fit to the training dataset was used to predict ischaemic stroke diagnosis using the validation dataset. The performance of this RNA-panel in predicting ischaemic stroke diagnosis was assessed using confusion matrices and receiver operator characteristic (ROC) curves.

### Sample size calculation

Previous case–control investigations using RNA-seq to compare between participants with ischaemic stroke or healthy controls have detected ≥ twofold differences in gene expression with as few as 3 individuals per group [8]. Anticipating greater inter-participant heterogeneity in gene expression in the current study, and the potential need to adjust regression analyses for confounders (up to 2 confounders based on previous analysis in this cohort) [51], we aimed to recruit at least 20 participants per group.

### Supplementary Information

**Additional file 1.** Supplementary data for the paper (Supplements 1–9).

## Data Availability

The datasets used and/or analysed during the current study are available from the corresponding author on reasonable request.
