# Supplementary material for: A case–control comparison of acute-phase peripheral blood gene expression in participants diagnosed with minor ischaemic stroke or stroke mimics
Source: Hum Genomics. 2023 Nov 25;17:106. doi: 10.1186/s40246-023-00551-y (PMC10676587; doi:10.1186/s40246-023-00551-y)
Supplement: Supplementary file 1 — Additional file 1. Supplementary data for the paper (Supplements 1–9). [file 40246_2023_551_MOESM1_ESM.pdf]

A case:control comparison of peripheral blood gene expression in participants presenting to hospital with ischaemic stroke or stroke mimics during the hyper-acute phase.

Joseph V Moxon, Andrew Calcino, Ann-Katrin Kraeuter, James Phie, Georgina Anderson, Glenys Standley, Cindy Sealey, Rhondda E. Jones, Matt A. Field, Jonathan Golledge

Supplementary data

Supplement 1: RNAs showing significant differences in expression between groups of participants with ischaemic stroke and stroke mimics (analysis of whole cohohort, n=56).

Supplement 2: miRNAs showing significant differences in expression between groups of participants with ischaemic stroke and stroke mimics (analysis of whole cohohort, n=56).

Supplement 3: Examination of selected protein coding genes suggested to be differentially expressed following the whole cohort analysis.

Supplement 4: RNAs showing significant differences in expression between groups of participants with ischaemic stroke and stroke mimics during the sensitivity analysis (n=54).

Supplement 5: miRNAs showing significant differences in expression between groups of participants with ischaemic stroke and stroke mimics during the sensitivity analysis (n=54).

Supplement 6: The association of differentially expressed genes with ischaemic stroke severity, symptom duration and TOAST classification

Supplement 7: Correlation of RNAs with targeting miRNAs which were significantly differentially expressed between groups of participants with ischaemic stroke or stroke mimics.

Supplement 8: Performance of models generated through machine learning models generated using CART, partial least squares- and RMSE regression and random forest analyses in predicting ischaemic stroke presence using the training data.

Supplement 9: Variable importance data detailing outputs from Random Forest analyses

Supplement 1: RNAs showing significant differences in expression between groups of participants with ischaemic stroke and stroke mimics (analysis of whole cohohort, n=56).

Gene ID	Gene Symbol	Gene Name	Mean expression in		Difference between groups		
			Ischaemic stroke	Stroke Mimics	LogFC	LogFC sd	p-value
ENSG00000144820	ADGRG7	Adhesion G protein-coupled receptor G7	0.49	3.46	-3.08	1.97	3.02E-08
ENSG00000118113	MMP8	Matrix metallopeptidase 8	4.14	45.40	-2.41	1.61	3.75E-05
ENSG00000211935	IGHV1-3	Immunoglobulin heavy variable 1-3	0.48	4.41	-2.26	1.45	5.23E-04
ENSG00000100448	CTSG	Cathepsin G	1.04	9.28	-2.17	1.40	5.23E-04
ENSG00000239839	DEFA3	Defensin alpha 3	56.65	529.76	-2.04	1.53	8.43E-04
ENSG00000173391	OLR1	Oxidized low density lipoprotein receptor 1	0.96	6.33	-1.86	1.33	1.41E-03
ENSG00000122641	INHBA	Inhibin subunit beta A	0.83	4.21	-1.79	1.35	1.48E-03
ENSG00000211623	IGKV2D-26	Immunoglobulin kappa variable 2D-26	0.03	0.89	-3.18	2.27	1.76E-03
ENSG00000133169	BEX1	Brain expressed X-linked 1	0.20	1.35	-2.00	1.16	1.76E-03
ENSG00000206047	DEFA1	Defensin alpha 1	88.05	694.59	-1.88	1.47	1.76E-03
ENSG00000240247	DEFA1B	Defensin alpha 1B	88.18	695.39	-1.88	1.47	1.76E-03
ENSG00000283646	ENSG00000283646	NA	1.28	6.67	-1.62	1.16	2.37E-03
ENSG00000148346	LCN2	Lipocalin 2	3.20	17.97	-1.65	1.18	3.13E-03
ENSG00000164821	DEFA4	Defensin alpha 4	2.87	23.23	-1.89	1.42	3.13E-03
ENSG00000281383	ENSG00000281383	NA	172.19	548.23	-1.86	1.46	3.67E-03
ENSG00000189182	KRT77	keratin 77	0.14	0.80	-2.76	1.40	5.09E-03
ENSG00000152760	TCTEX1D1	Tctex1 domain containing 1	0.58	2.69	-1.58	1.22	6.73E-03
ENSG00000280614	ENSG00000280614	NA	415.58	1175.39	-1.73	1.35	6.73E-03
ENSG00000280800	ENSG00000280800	NA	415.48	1174.04	-1.73	1.35	6.73E-03
ENSG00000281181	ENSG00000281181	NA	415.54	1176.04	-1.73	1.35	6.73E-03
ENSG00000227502	ENSG00000227502	NA	0.12	0.61	-1.62	1.12	7.07E-03
ENSG00000197561	ELANE	Elastase, neutrophil expressed	1.00	6.25	-1.68	1.37	7.41E-03
ENSG00000162437	RAVER2	Ribonucleoprotein, PTB binding 2	4.98	11.18	-0.99	0.34	8.26E-03
ENSG00000223518	ENSG00000223518	NA	0.09	0.66	-2.97	2.19	8.26E-03
ENSG00000254006	ENSG00000254006	NA	0.99	4.42	-1.50	1.14	9.06E-03
ENSG00000167306	MYO5B	Myosin VB	2.96	4.88	-1.09	0.29	1.04E-02
ENSG00000169397	RNASE3	Ribonuclease A family member 3	0.77	4.18	-1.55	1.28	1.19E-02

ENSG00000157554	ERG	ERG, ETS transcription factor	4.23	11.70	-1.30	0.85	1.23E-02
ENSG00000188761	BCL2L15	BCL2 like 15	0.91	2.85	-1.21	0.72	1.23E-02
ENSG00000172232	AZU1	Azurocidin 1	0.71	2.92	-1.47	1.03	1.34E-02
ENSG00000005381	MPO	Myeloperoxidase	3.03	12.02	-1.31	1.00	1.64E-02
ENSG00000065618	COL17A1	Collagen type XVII alpha 1 chain	0.69	2.44	-1.51	1.26	2.05E-02
ENSG00000124469	CEACAM8	Carcinoembryonic antigen related cell adhesion molecule 8	2.88	11.62	-1.33	1.00	2.12E-02
ENSG00000243144	ENSG00000243144	NA	1.76	4.56	-1.41	0.89	2.62E-02
ENSG00000111339	ART4	ADP-ribosyltransferase 4 (Dombrock blood group)	0.38	1.24	-1.28	0.98	2.62E-02
ENSG00000248385	TARM1	T cell-interacting, activating receptor on myeloid cells 1	0.17	0.57	-1.44	0.98	2.62E-02
ENSG00000224940	PRRT4	Proline rich transmembrane protein 4	0.19	0.47	-1.34	0.64	2.62E-02
ENSG00000137675	MMP27	Matrix metallopeptidase 27	0.05	0.18	-2.45	1.44	2.62E-02
ENSG00000096006	CRISP3	Cysteine rich secretory protein 3	3.19	13.38	-1.41	0.96	2.63E-02
ENSG00000236333	ENSG00000236333	NA	0.37	1.20	-1.48	0.64	3.14E-02
ENSG00000174358	SLC6A19	Solute carrier family 6 member 19	2.17	0.60	1.49	1.19	3.45E-02
ENSG00000163710	PCOLCE2	Procollagen C-endopeptidase enhancer 2	0.74	1.87	-1.33	1.06	3.45E-02
ENSG00000149516	MS4A3	Membrane spanning 4-domains A3	3.92	16.20	-1.27	1.12	3.45E-02
ENSG00000006740	ARHGAP44	Rho GTPase activating protein 44	1.44	2.72	-1.22	0.75	4.15E-02
ENSG00000101425	BPI	Bactericidal permeability increasing protein	2.90	9.17	-1.21	0.85	4.15E-02
ENSG00000272398	CD24	CD24 molecule	14.71	48.07	-1.14	0.69	4.24E-02

Expression values refer to counts per million for that transcript. Log FC: Fold change in transcript expression in participants with strokes, compared to stroke mimics. Std Dev: Standard deviation of fold change. P-value refers to False Discovery Rate corrected ‘P-union’ p-value reported by consensusDE. NA: not annotated.

Supplement 2: miRNAs showing significant differences in expression between groups of participants with ischaemic stroke and stroke mimics (analysis of whole cohohort, n=56).

Gene ID	Gene Symbol	Gene Family *	Mean expression in		Difference between groups		
			Ischaemic stroke	Stroke Mimics	LogFC	LogFC sd	p-value
ENSG00000198974	MIR30E	mir-30	3613.71	3097.52	0.32	0.07	9.00E-04
ENSG00000265565	MIR3143	NA	9.31	7.73	0.41	0.08	1.06E-02
ENSG00000276830	MIR6730	NA	2.68	3.9	-0.52	0.06	1.41E-02
ENSG00000284464	MIR1306	mir-1306	94.96	129.45	-0.38	0.07	1.43E-02
ENSG00000283450	MIR486-2	mir-486	63.61	86.84	-0.43	0.08	1.43E-02
ENSG00000276926	MIR6797	NA	1.21	1.85	-0.55	0.06	1.43E-02
ENSG00000221598	MIR1249	mir-1249	11.13	15.78	-0.52	0.08	1.68E-02
ENSG00000207952	MIR624	mir-624	27.83	22.23	0.55	0.07	1.93E-02
ENSG00000283935	MIR423	mir-423	2654.16	3604.66	-0.33	0.07	1.99E-02
ENSG00000264610	MIR4685	NA	83.12	113.12	-0.39	0.07	1.99E-02
ENSG00000199168	MIR374A	mir-374	239.35	187.26	0.6	0.07	2.02E-02
ENSG00000271886	MIR98	let-7	290.66	212.71	0.5	0.08	2.75E-02
ENSG00000207948	MIR328	mir-328	462.12	595.79	-0.33	0.07	2.95E-02
ENSG00000274705	MIR486-1	mir-486	664.64	856.84	-0.35	0.08	3.10E-02
ENSG00000265483	MIR4443	NA	5.2	10.4	-0.8	0.19	3.32E-02
ENSG00000275101	MIR6766	NA	2.76	3.74	-0.42	0.08	3.59E-02
ENSG00000207758	MIR532	mir-188	2638.72	3236.98	-0.25	0.07	3.61E-02
ENSG00000199122	MIR148B	mir-148	2024.2	1853.78	0.21	0.07	4.02E-02
ENSG00000283785	MIR15A	mir-15	2103.82	1567.99	0.53	0.09	4.09E-02
ENSG00000212027	MIR374B	mir-374	303.49	251.15	0.49	0.07	4.09E-02
ENSG00000199158	MIR96	mir-96	1128.39	811.36	0.56	0.09	4.09E-02
ENSG00000266297	MIR744	mir-744	251.89	311.89	-0.26	0.07	4.14E-02
ENSG00000207698	MIR32	mir-32	41.24	23.06	0.92	0.07	4.42E-02
ENSG00000275519	MIR6804	NA	2.18	3.11	-0.43	0.07	4.45E-02
ENSG00000283762	MIR20A	mir-17	494.21	339.66	0.55	0.12	4.76E-02
ENSG00000207864	MIR27B	mir-27	340.31	281.18	0.38	0.07	4.76E-02
ENSG00000198995	MIR340	mir-340	159.82	138.48	0.27	0.07	4.76E-02
ENSG00000211514	MIR454	mir-454	1153.58	987.41	0.32	0.08	4.76E-02
ENSG00000264157	MIR3127	mir-3127	5.42	7.05	-0.3	0.08	4.81E-02
ENSG00000273898	MIR6798	NA	3.23	4.32	-0.39	0.09	4.95E-02

miRNA gene family annotation based on assignments published in miRbase. Expression values refer to counts per million for that transcript. Log FC: Fold change in transcript expression in participants with strokes, compared to stroke mimics. Std Dev: Standard deviation of fold change. P-value refers to False Discovery Rate corrected ‘P-union’ p-value reported by consensusDE. NA: not annotated.

Supplement 3: Examination of selected protein coding genes suggested to be differentially expressed following the whole cohort analysis.

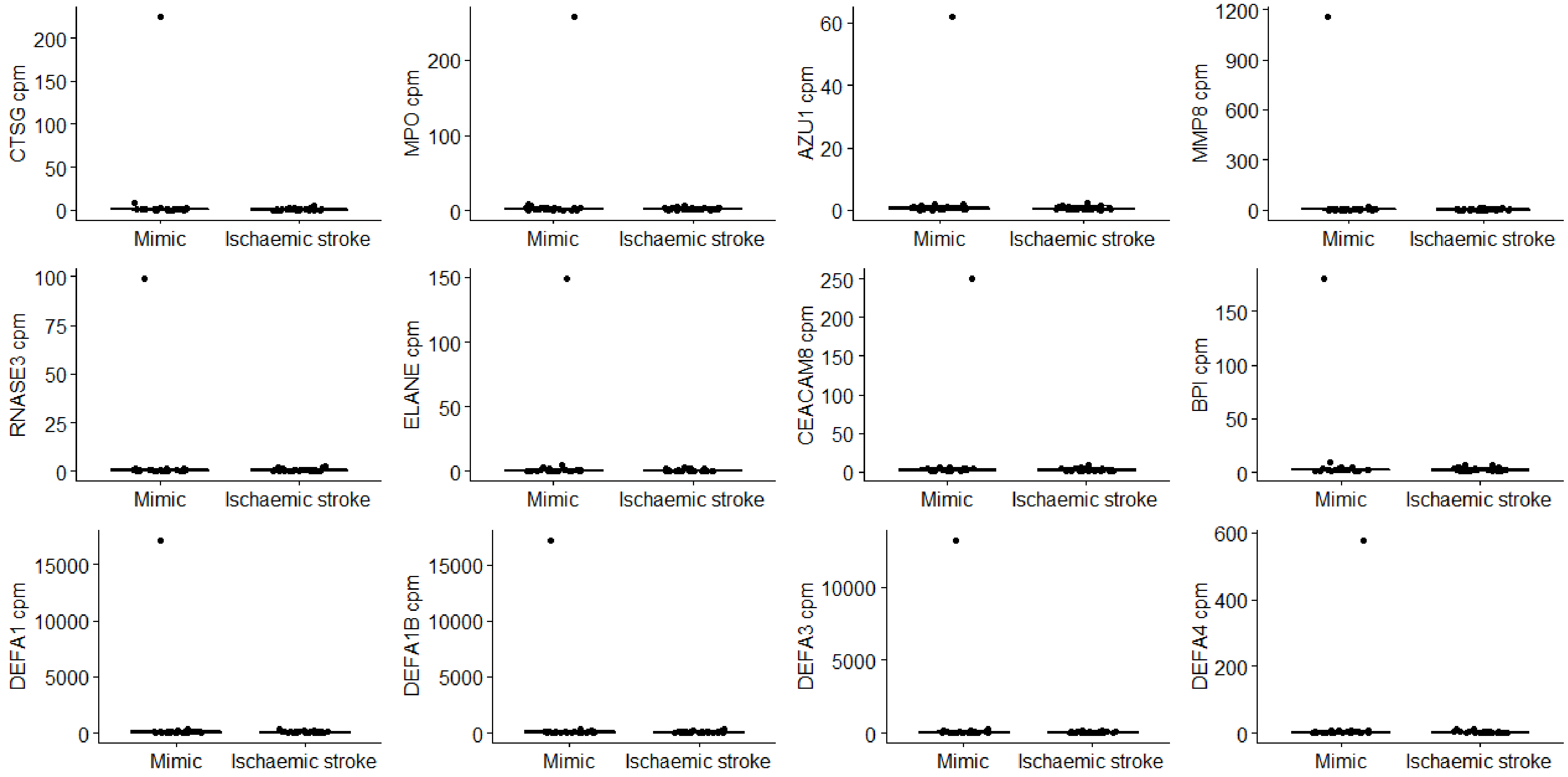

Plots show protein coding gene expression as normalized fragment counts per million (CPM). A clear outlier is seen in the stroke mimic group representing an individual with metastatic disease

Supplement 4: RNAs showing significant differences in expression between groups of participants with ischaemic stroke and stroke mimics during the sensitivity analysis (n=54).

Gene ID	Gene Symbol	Gene Name	Mean expression in		Difference between groups		
			Ischaemic stroke	Stroke Mimics	LogFC	LogFC sd	p-value
ENSG00000144820	ADGRG7	Adhesion G protein-coupled receptor G7	0.48	3.73	-3.22	1.96	6.90E-09
ENSG00000211935	IGHV1-3	Immunoglobulin heavy variable 1-3	0.48	4.74	-2.35	1.42	6.14E-04
ENSG00000211623	IGKV2D-26	Immunoglobulin kappa variable 2D-26	0.03	0.95	-3.23	2.32	6.75E-03
ENSG00000281383	ENSG00000281383	NA	172.20	587.10	-1.96	1.49	8.02E-03
ENSG00000167306	MYO5B	Myosin VB	2.96	5.22	-1.21	0.27	8.24E-03
ENSG00000280800	ENSG00000280800	NA	415.51	1255.29	-1.82	1.38	9.80E-03
ENSG00000280614	ENSG00000280614	NA	415.60	1256.74	-1.82	1.38	9.80E-03
ENSG00000281181	ENSG00000281181	NA	415.57	1257.44	-1.82	1.38	9.80E-03
ENSG00000223518	ENSG00000223518	NA	0.09	0.70	-3.06	2.24	1.85E-02
ENSG00000162437	RAVER2	Ribonucleoprotein, PTB binding 2	4.98	11.44	-1.01	0.35	2.59E-02

Expression values refer to counts per million for that transcript. Log FC: Fold change in transcript expression in participants with strokes, compared to stroke mimics. Std Dev: Standard deviation of fold change. P-value refers to False Discovery Rate corrected ‘P-union’ p-value reported by consensusDE. NA: not annotated.

Supplement 5: miRNAs showing significant differences in expression between groups of participants with ischaemic stroke and stroke mimics during the sensitivity analysis (n=54).

Gene ID	Gene Symbol	Gene Family	Mean expression in		Difference between groups		
			Ischaemic stroke	Stroke Mimics	LogFC	LogFC sd	p-value
ENSG00000198974	MIR30E	mir-30	3613.74	3069.86	0.35	0.09	4.36E-05
ENSG00000276830	MIR6730	NA	2.67	4.08	-0.6	0.08	8.45E-04
ENSG00000284464	MIR1306	mir-1306	94.97	134.34	-0.44	0.09	1.11E-03
ENSG00000265565	MIR3143	NA	9.3	7.67	0.44	0.09	2.34E-03
ENSG00000283450	MIR486-2	mir-486	63.61	89.2	-0.47	0.09	3.47E-03
ENSG00000276926	MIR6797	NA	1.21	1.91	-0.6	0.07	3.47E-03
ENSG00000221598	MIR1249	mir-1249	11.12	16.22	-0.56	0.09	5.38E-03
ENSG00000207948	MIR328	mir-328	462.13	611.93	-0.37	0.08	7.56E-03
ENSG00000274705	MIR486-1	mir-486	664.65	881.75	-0.39	0.09	7.62E-03
ENSG00000264610	MIR4685	NA	83.12	114.93	-0.41	0.08	7.62E-03
ENSG00000273898	MIR6798	NA	3.22	4.53	-0.47	0.1	7.62E-03
ENSG00000275101	MIR6766	NA	2.76	3.88	-0.48	0.09	7.62E-03
ENSG00000207758	MIR532	mir-188	2638.74	3318.02	-0.28	0.08	7.81E-03
ENSG00000283935	MIR423	mir-423	2654.18	3667.31	-0.34	0.08	7.81E-03
ENSG00000199168	MIR374A	mir-374	239.36	182.91	0.66	0.08	8.20E-03
ENSG00000274111	MIR6777	NA	5.93	8.31	-0.4	0.09	9.22E-03
ENSG00000207952	MIR624	mir-624	27.83	22.15	0.57	0.08	1.03E-02
ENSG00000216001	MIR450B	mir-450	2.76	1.74	0.78	0.13	1.04E-02
ENSG00000283785	MIR15A	mir-15	2103.84	1534.94	0.58	0.1	1.04E-02
ENSG00000221630	MIR1179	mir-1179	4.22	2.93	0.57	0.09	1.04E-02
ENSG00000212027	MIR374B	mir-374	303.48	239.63	0.54	0.08	1.04E-02
ENSG00000199122	MIR148B	mir-148	2024.21	1847.26	0.23	0.08	1.04E-02
ENSG00000275519	MIR6804	NA	2.17	3.23	-0.5	0.08	1.05E-02
ENSG00000273555	MIR6812	NA	5.38	7.49	-0.38	0.08	1.14E-02
ENSG00000207698	MIR32	mir-32	41.25	22.2	1.01	0.08	1.34E-02
ENSG00000263816	MIR548AW	mir-548	1.99	1.3	0.65	0.13	1.34E-02
ENSG00000271886	MIR98	let-7	290.66	215.89	0.49	0.09	1.34E-02
ENSG00000284190	MIR21	mir-21	766.79	490.53	0.67	0.11	1.37E-02
ENSG00000283762	MIR20A	mir-17	494.22	333.97	0.59	0.13	1.37E-02
ENSG00000211514	MIR454	mir-454	1153.59	995.98	0.33	0.09	1.37E-02
ENSG00000198995	MIR340	mir-340	159.81	138.44	0.29	0.08	1.37E-02
ENSG00000199158	MIR96	mir-96	1128.4	803.98	0.59	0.1	1.51E-02
ENSG00000284163	MIR3620	NA	3.71	5.08	-0.38	0.08	1.55E-02
ENSG00000198997	MIR107	mir-103	25.93	20.43	0.41	0.08	1.58E-02
ENSG00000275640	MIR6793	NA	4.19	5.58	-0.36	0.08	1.65E-02
ENSG00000207944	MIR574	mir-574	472.01	639.96	-0.32	0.09	1.80E-02
ENSG00000265483	MIR4443	NA	5.21	10.44	-0.73	0.22	1.87E-02
ENSG00000283840	MIR6764	NA	4.48	5.56	-0.27	0.08	1.90E-02
ENSG00000284519	MIR6741	NA	16.22	21.06	-0.3	0.09	1.91E-02
ENSG00000263892	MIR4667	mir-4667	1.61	2.36	-0.5	0.07	1.98E-02
ENSG00000283805	MIR636	mir-636	79.2	98.89	-0.28	0.08	2.03E-02
ENSG00000199065	MIR101-2	mir-101	49.54	30.85	0.68	0.18	2.09E-02
ENSG00000264607	MIR3173	mir-3173	231.7	294.6	-0.28	0.08	2.20E-02
ENSG00000207628	MIR651	mir-651	2.77	1.79	0.78	0.09	2.22E-02

ENSG00000284182	MIR143	mir-143	81.51	54.33	0.6	0.12	2.22E-02
ENSG00000207864	MIR27B	mir-27	340.31	282.54	0.39	0.08	2.22E-02
ENSG00000284499	MIR363	mir-363	2023.83	1656.77	0.33	0.09	2.22E-02
ENSG00000207947	MIR152	mir-148	72.63	62.6	0.29	0.08	2.22E-02
ENSG00000207980	MIR23A	mir-23	2164.06	1950.27	0.19	0.09	2.22E-02
ENSG00000266297	MIR744	mir-744	251.88	313.93	-0.26	0.08	2.42E-02
ENSG00000199150	MIRLET7G	let-7	18258.42	13384.46	0.56	0.08	2.50E-02
ENSG00000264157	MIR3127	mir-3127	5.42	7.13	-0.3	0.09	2.57E-02
ENSG00000276102	MIR6747	NA	53.4	70.19	-0.35	0.08	2.76E-02
ENSG00000221410	MIR1238	mir-1238	3.3	4.49	-0.37	0.1	2.94E-02
ENSG00000207708	MIR141	mir-8	1.82	1.1	0.8	0.08	3.01E-02
ENSG00000199121	MIR26B	mir-26	7540.46	5895.93	0.5	0.07	3.05E-02
ENSG00000223109	MIR1538	mir-1538	1.16	1.65	-0.43	0.07	3.12E-02
ENSG00000207996	MIR301A	mir-130	26.18	20.04	0.55	0.09	3.17E-02
ENSG00000278851	MIR6879	NA	6.47	8.07	-0.28	0.08	3.26E-02
ENSG00000284443	MIR197	mir-197	142.71	183.24	-0.3	0.09	3.27E-02
ENSG00000283604	MIR338	mir-338	26.63	21.04	0.39	0.1	3.30E-02
ENSG00000283522	MIR3150A	mir-3150	9.3	11.42	-0.22	0.09	3.39E-02
ENSG00000274417	MIR6515	NA	16.56	20.96	-0.3	0.08	3.39E-02
ENSG00000278349	MIR6754	NA	6.01	8	-0.39	0.09	3.39E-02
ENSG00000278359	MIR7155	NA	1.59	2.39	-0.48	0.08	3.39E-02
ENSG00000283891	MIR628	mir-628	57.93	50.08	0.26	0.08	3.59E-02
ENSG00000277474	MIR6894	NA	3.82	5.14	-0.44	0.09	3.65E-02
ENSG00000208002	MIR643	mir-643	6.34	5.22	0.42	0.08	3.82E-02
ENSG00000203709	MIR29B2CHG	NA	963.69	826.35	0.27	0.08	4.09E-02
ENSG00000278328	MIR6802	NA	49.08	59	-0.25	0.08	4.25E-02
ENSG00000284214	MIR29C	mir-29	913.59	774.64	0.29	0.08	4.49E-02
ENSG00000284186	MIR3615	mir-3615	1727.96	2184.91	-0.32	0.09	4.55E-02
ENSG00000276869	MIR6729	NA	1.56	2.16	-0.4	0.07	4.55E-02
ENSG00000264500	MIR3124	NA	7.05	8.85	-0.26	0.09	4.92E-02

miRNA gene family annotation based on assignments published in miRbase. Expression values refer to counts per million for that transcript. Log FC: Fold change in transcript expression in participants with strokes, compared to stroke mimics. Std Dev: Standard deviation of fold change. P-value refers to False Discovery Rate corrected ‘P-union’ p-value reported by consensusDE. NA: not annotated.

Supplement 6: The association of differentially expressed genes with ischaemic stroke severity, symptom duration and TOAST classification

Gene	NIHSS score			Symptom duration			TOAST	
	Spearman's rho	P-value	Q-value	Spearman's rho	P-value	Q-value	P-value	Q-value
ADGRG7	0.123	0.587	0.903	0.107	0.604	0.924	0.297	0.495
ENSG00000223518	-0.357	0.103	0.729	0.065	0.751	0.924	0.050	0.347
ENSG00000280614	-0.172	0.445	0.800	0.335	0.095	0.523	0.658	0.711
ENSG00000280800	-0.172	0.445	0.800	0.335	0.095	0.523	0.658	0.711
ENSG00000281181	-0.172	0.445	0.800	0.335	0.095	0.523	0.658	0.711
ENSG00000281383	-0.219	0.335	0.800	0.312	0.121	0.539	0.546	0.682
IGHV1-3	-0.038	0.865	0.968	0.030	0.884	0.947	0.478	0.638
IGKV2D-26	-0.467	0.028	0.567	-0.103	0.616	0.924	0.788	0.808
MYO5B	-0.115	0.610	0.904	0.089	0.664	0.924	0.007	0.251
RAVER2	-0.408	0.059	0.729	0.405	0.040	0.523	0.056	0.347
MIR15A	-0.014	0.952	0.976	0.085	0.681	0.924	0.563	0.682
MIR20A	-0.101	0.655	0.904	0.071	0.730	0.924	0.461	0.636
MIR27B	0.086	0.702	0.904	0.026	0.900	0.947	0.369	0.567
MIR30E	0.063	0.781	0.946	0.062	0.762	0.924	0.155	0.442
MIR32	0.182	0.419	0.800	0.366	0.066	0.523	0.138	0.442
MIR96	-0.510	0.015	0.567	0.026	0.900	0.947	0.896	0.896
MIR98	-0.205	0.360	0.800	-0.041	0.842	0.947	0.271	0.470
MIR148B	-0.219	0.327	0.800	-0.437	0.026	0.523	0.270	0.470
MIR328	0.182	0.419	0.800	0.366	0.066	0.523	0.138	0.442
MIR340	0.003	0.990	0.990	-0.032	0.876	0.947	0.188	0.442
MIR374A	0.032	0.887	0.968	-0.009	0.966	0.970	0.435	0.622
MIR374B	-0.220	0.324	0.800	-0.127	0.536	0.924	0.177	0.442
MIR423	0.335	0.128	0.729	0.118	0.567	0.924	0.086	0.428
MIR454	-0.145	0.520	0.867	0.008	0.970	0.970	0.224	0.465
MIR486-1	0.383	0.078	0.729	0.258	0.204	0.749	0.061	0.347
MIR486-2	0.336	0.127	0.729	0.117	0.570	0.924	0.041	0.347
MIR532	0.166	0.460	0.800	0.204	0.318	0.749	0.206	0.459
MIR624	-0.101	0.654	0.904	-0.064	0.756	0.924	0.533	0.682
MIR744	0.175	0.435	0.800	0.326	0.105	0.523	0.244	0.465
MIR1249	0.250	0.261	0.800	0.099	0.629	0.924	0.120	0.442
MIR1306	0.282	0.203	0.800	0.168	0.413	0.869	0.383	0.567
MIR3127	0.216	0.335	0.800	0.217	0.286	0.749	0.233	0.465
MIR3143	-0.025	0.913	0.968	-0.228	0.263	0.749	0.703	0.740
MIR4443	0.080	0.723	0.904	-0.226	0.266	0.749	0.048	0.347
MIR4685	0.243	0.275	0.800	0.179	0.382	0.848	0.177	0.442
MIR6730	0.135	0.549	0.878	0.225	0.269	0.749	0.341	0.546
MIR6766	0.090	0.691	0.904	0.204	0.318	0.749	0.166	0.442
MIR6797	0.052	0.818	0.962	0.255	0.208	0.749	0.155	0.442
MIR6798	-0.023	0.919	0.968	0.087	0.674	0.924	0.013	0.251
MIR6804	-0.224	0.317	0.800	0.072	0.727	0.924	0.618	0.711

P-values relate to raw values from initial statistical analysis, Q-values refer to P-values corrected for multiple testing using the Benjamini-Hochberg method. Symptom duration refers to the period between approximate symptom onset and presentation to hospital.

Supplement 7: Correlation of RNAs with targeting miRNAs which were significantly differentially expressed between groups of participants with ischaemic stroke or stroke mimics.

**RNA**	**mirDB predictions**			**Correlation between miRNA and target RNA expression**	
	**Interacting miRNA**	**Target score**	**Rank**	**Spearman's *ρ***	**p-value**
MYO5B	MIR15A [MIR15A-5p]	89	13/189	-0.120	0.535
	MIR374A [MIR374A-5p]	87	17/189	0.070	0.718
	MIR374B [MIR374B-5p]	85	26/189	-0.397	0.034
	MIR20A [MIR20A-5p]	78	51/189	-0.063	0.745
	MIR624 [MIR624-5p]	65	90/189	-0.058	0.766
	MIR96 [MIR96-5p]	61	110/189	0.016	0.934
	MIR98 [MIR98-5p]	53	160/189	-0.167	0.386
RAVER2	MIR532 [MIR532-5p]	94	3/142	0.136	0.479
	MIR30E [MIR30E-5p]	83	31/142	0.016	0.934
	MIR454 [MIR454-5p]	71	65/142	0.164	0.395
	MIR15A [MIR15A-3p]	70	67/142	0.054	0.782
	MIR96 [MIR96-5p]	58	107/142	-0.017	0.932
	MIR374A [MIR374A-5p]	52	125/142	0.012	0.952

Correlations calculated using normalized log-transformed counts per million data for the transcripts. Analyses conducted using data from the participants with ischaemic stroke only (n=29).

Supplement 8: Performance of classifier models in predicting ischaemic stroke presence.

Model	Accuracy % (95% CI)	p-value	Sensitivity (%)	Specificity (%)	PPV (%)	NPV (%)
Models run using the training dataset						
Partial least squares regression (5-fold cross validated, 100 repeats) [1]	84.9 (68.1 – 94.9)	2.44×10^{-4}	80.0	88.9	85.7	84.2
Root mean-square error regression (5-fold cross validated) [2]	84.9 (68.1 – 94.9)	2.44×10^{-4}	86.7	83.3	81.3	88.2
Random Forest (equal performance for 5 models containing 1000 – 3,000 trees) [3]	100 (89.4 – 100)	2.06×10^{-9}	100	100	100	100
Long-RNA and miRNA panel*	75.8 (57.7 – 88.9)	0.010	66.7	83.3	76.9	75.0
Models run using the validation dataset						
Long-RNA and miRNA panel*	47.6 (25.7 – 70.2)	0.999	40.0	50.0	20.0	72.7

Data generated using confusion matrices to assess model performance. P-value compares accuracy of model to the no information rate; PPV: positive predictive value; NPV: Negative predictive value.

*The RNA panel comprises ADGRG7, and miRs 96, 532, 6766, 6798, and 6804.

R package citations

1. Kuhn, M., *Building Predictive Models in R Using the caret Package.* J Stat Softw, 2008. **28**(5): 1-26.
2. Friedman, J., T. Hastie, & R. Tibshirani, *Regularization Paths for Generalized Linear Models via Coordinate Descent.* J Stat Softw, 2010. **33**(1): 1-22.
3. Liaw, A. & M. Wiener, *Classification and Regression by randomForest.* R News, 2002. **2**(3): 18-22.

Supplement 9: Variable importance data detailing outputs from Random Forest analyses

Gene	Mean decrease in accuracy scores for each model				
	1000 trees	1500 trees	2000 trees	2500 trees	3000 trees
ADGRG7	7.5364	7.5344698	9.80348	9.9312669	12.9419472
IGHV1.3	-0.6461	-1.1388167	-0.378135	1.8480461	-0.5004014
IGKV2D.26	0.3240	-0.9072147	-0.794282	0.1904545	-0.267148
E281383	-3.1397	-3.5293574	-3.603502	-4.058517	-4.0719566
MYO5B	-0.7782	1.1120417	1.0742957	1.6302383	-0.228696
E280800	-1.3808	-3.4399727	-1.947968	-3.8936813	-4.5167581
E280614	-0.7509	-1.7633503	-2.748354	-3.6601087	-3.8038704
E281181	0.2229	-2.8515754	-3.964395	-3.7937458	-3.5023867
E223518	-0.2775	-1.4828929	-0.470846	-2.6799793	0.16912389
RAVER2	1.6605	0.6353827	2.148159	2.0280594	2.57839845
MIR30E	2.1107	3.934026	3.2280433	4.8324339	4.24550238
MIR6730	-0.6372	3.1525505	5.725832	3.9151859	6.18282437
MIR1306	-0.7231	-2.2699582	-1.929684	-0.1494321	-2.8311329
MIR3143	-0.9969	-0.9863566	-1.30588	-1.7702533	-3.5330134
MIR486.2	2.6341	0.1910546	1.7287171	0.029325	-0.3594068
MIR6797	3.1821	0.8045639	2.253921	4.7280451	2.73965508
MIR1249	0.4737	-0.0585157	-0.719306	-1.431432	-0.6139627
MIR328	-2.4691	-2.2902994	-2.136251	-4.3353033	-3.7416502
MIR486.1	-0.1279	-0.9484341	0.4943274	-3.1520196	-1.3589216
MIR4685	2.1367	2.9304605	3.8567702	3.8624737	2.0586721
MIR6766	3.7262	4.4786353	5.8639477	4.4614579	6.80241441
MIR6798	7.1982	6.5762617	8.5301302	11.32793	12.7183739
MIR423	-0.6794	-1.9566305	-1.875213	-1.4649312	-0.9847005
MIR532	6.0095	7.8454985	8.1839118	10.535461	9.55609265
MIR374A	-1.0207	-0.3035988	0.1175179	-2.9459462	-1.6156573
MIR624	-1.7027	-0.5043064	-2.307472	-1.5497355	-1.0153279
MIR15A	-1.8017	-2.8992823	-2.361025	-0.7032724	0.39411213
MIR148B	-2.4621	-1.4220461	-0.673654	-3.0319248	-4.9532518
MIR374B	-1.6331	0.2908325	-2.970528	-3.201003	-2.0278113
MIR6804	5.8921	7.2039155	7.0561507	8.270503	10.6926339
MIR98	-2.7045	-2.1581023	-2.790409	-2.6048826	-4.0695872
MIR20A	-0.2738	-2.2387321	-2.191866	-3.3328861	-2.7987084
MIR340	0.3819	-0.3475546	0.2331554	0.1954979	3.67902274
MIR454	-0.5153	0.321155	-2.729762	-0.6096476	0.00479666
MIR96	7.3406	8.9713818	12.531603	13.332779	14.4123348
MIR4443	0.9652	1.1945863	0.4485399	2.8057858	1.10749552
MIR27B	-1.0512	-2.858139	-1.770446	-1.989453	-0.513237
MIR744	-0.7291	2.0110653	0.451536	-2.630022	-0.5581427
MIR3127	-2.5545	-2.3682147	-3.301821	-3.781653	-4.501413
MIR32	-3.0079	-1.8323645	-0.184794	0.2933296	-1.6059659

Red shaded cells: Genes with mean decrease in accuracy scores between 1 and 2;
Yellow shaded cells: Genes with mean decrease in accuracy scores between 2 and 3;
Green shaded cells: Genes with mean decrease in accuracy scores between >3;

Genes showing mean decrease in accuracy scores >3 for all models were selected as for assessment of diagnostic potential in binary logistic regression models on the validation dataset.
